# Colour Constancy Across the Life Span: Evidence for Compensatory Mechanisms

**DOI:** 10.1371/journal.pone.0063921

**Published:** 2013-05-08

**Authors:** Sophie Wuerger

**Affiliations:** Department of Psychological Sciences, Institute of Psychology, Health, and Society, University of Liverpool, United Kingdom; University of Sussex, United Kingdom

## Abstract

It is well known that the peripheral visual system declines with age: the yellowing of the lens causes a selective reduction of short-wavelength light and sensitivity losses occur in the cone receptor mechanisms. At the same time, our subjective experience of colour does not change with age. The main purpose of this large-scale study (n = 185) covering a wide age range of colour-normal observers (18–75 years of age) was to assess the extent to which the human visual system is able to compensate for the changes in the optical media and at which level of processing this compensation is likely to occur. We report two main results: (1) Supra-threshold parafoveal colour perception remains largely unaffected by the age-related changes in the optical media (yellowing of the lens) whereas our ability to discriminate between small colour differences is compromised with an increase in age. (2) Significant changes in colour appearance are only found for unique green settings under daylight viewing condition which is consistent with the idea that the yellow-blue mechanism is most affected by an increase in age due to selective attenuation of short-wavelength light. The data on the invariance of hue perception, in conjunction with the age-related decline in chromatic sensitivity, provides evidence for compensatory mechanisms that enable colour-normal human observers a large degree of colour constancy across the life span. These compensatory mechanisms are likely to originate at cortical sites.

## Introduction

The purpose of this study was to assess in a large sample of adult colour-normal observers whether higher-order colour appearance mechanisms are affected by ageing. Human colour vision starts in the retinal photoreceptors, where the lights is absorbed by the long-, medium- and short-wavelength-sensitive (L-, M-, or S-) cones [1]. The cone outputs are recombined in post-receptoral cone-opponent retinal channels and their spectral tuning is inherited by the Lateral Geniculate Nucleus (LGN), a sub-cortical visual relay station [2]. These early cone-opponent mechanisms feed into cortical colour mechanisms with different spectral properties that have not been fully characterised [3]–[5]. The cortical colour mechanisms that mediate the unique hues have been characterised using behavioural methods [6]–[11], but the neural basis of the unique hues is still an unresolved issue [12], [13]. In our study we use unique hue judgements as a way to gauge higher-order colour appearance mechanisms since unique hue judgments allow us to obtain absolute appearance judgements without the need for a reference.

Previous studies suggest that hue perception is fairly constant across the life span [14], [15]. At the same time age-related changes in the optical media (yellowing of the lens; [16]) occur; the number of retinal ganglion cells is likely to decrease with age [17], at the cortical level losses in dendritic spines and changes in the morphology of myelin sheathes and axons lead to a decrease in neural efficiency [18]. A possible explanation for hue constancy with an increase in age is that the higher-order colour mechanisms that mediate hue perception take the difference between the cone signals and are therefore not greatly affected by a signal loss in the peripheral system [14]. For example, an L-M mechanism will stay roughly constant with age, if the decline in the L and M cone signal is parallel. Here we test this hypothesis directly by assessing hue perception in a large-scale study (n = 185) for observers spanning a wide age range (18–75 years of age) and then compare the hue settings with the prediction derived from the changes in the optical media. Our second aim was to evaluate whether adaptation mechanisms that ensure constancy across different ambient viewing conditions, are affected by age [17]. We find that, to a large extent, hue perception is invariant with age; the direction but not the magnitude of the small observed age-related hue changes are predicted by the yellowing of the lens. Our results are consistent with compensatory mechanisms that operate on higher-order colour vision but not on early colour discrimination mechanisms.

## Methods

We report a series of three experiments conducted with the same set of colour-normal observers (n = 185) covering a wide age range (18–75 y.o.a). All observers made settings under three different ambient illumination conditions, with each condition being tested on a different occasion: under dark viewing conditions, under a day-light simulator (close to D65), or under typical cool white office lighting (CWF).

### Equipment

Stimuli were displayed on CRT monitor (21-inch Sony GDM-F520) which was controlled by a DELL PC with a ViSaGe stimulus generator (Cambridge Research System, Ltd.). The lookup tables were linearised using the ColourCal calibration device (Cambridge Research System, Ltd.) which interfaces with the graphics card. Calibration was checked with a PR650 tele-spectroradiometer (PhotoResearch). The CRT monitor had a correlated colour temperature of 9300K with a peak luminance of 120 cd/m^2^. The CIE coordinates (x, y, Luminance) of the phosphors at peak output (measured in an otherwise dark room) were as follows: red = 0.627, 0.342, 28.12; green = 0.287, 0.608, 80.96; blue = 0.151, 0.074, 14.16, respectively. The background was always set to a mid-grey (CIE x = 0.2897, y = 0.2977) with a luminance of 23.9 cd/m^2^. Since there was some initial monitor drift, the monitor was switched on at least one hour before the start of the experiment. The responses of the observers were collected using a button box (CT6, Cambridge Research System, Ltd.). Stimuli were generated using the CRS MatLab toolbox and MatLab 7.4.

The experiments were conducted in a sound-attenuated booth the ceiling of which was equipped with a GTI ColorMatcher GLE M5/25 to generate two ambient lighting conditions: a D65 simulator for daylight and CWF for typical office light. The inside of the booth (2 m×2 m) is dark grey reflecting only a small amount of light. A white tile (100% reflectance) was placed underneath the light sources and measured by a PhotoResearch PR-650 telespectroradiometer (TSR). Their specifications (CIE xyLum and correlated colour temperature) are as follows; for D65: luminance = 41.3 cd/m^2^; x = 0.3229; y = 0.3453; CCT = 5917; for CWF: luminance = 136.8 cd/m^2^; x = 0.3890; y = 0.3887; CCT = 3866.

Since the light reflected from the monitor screen is to a small extent affected by the prevailing illumination, the RGB outputs of the monitor were measured with the spectroradiometer (PR650) under all three illumination conditions and the L,M,S values were computed separately for each of the viewing conditions.

It is likely that observers were adapted to both, the prevailing illumination and to the monitor background; however, since the monitor background was kept constant for all viewing conditions, any observed difference in the unique hue settings must be attributed to the prevailing illumination.

### Subjects

185 (82 males and 103 females) naïve subjects participated in the experiment, with a mean age of 34.03 years (range: 18–75 years). Subjects were paid and informed consent was obtained from all subjects prior to the experiment. All subjects had normal or corrected to normal visual acuity; subjects with any history of cataracts or any other eye surgery were excluded from the experiment. Written consent was obtained from each participant prior to the study. The experiments have been approved by the Ethics committee in the Institute of Psychology, Health, and Society, at the University of Liverpool, UK. All experiments are in accordance with the principles expressed in the Declaration of Helsinki.

### Stimuli and procedure

**Figure 1:**
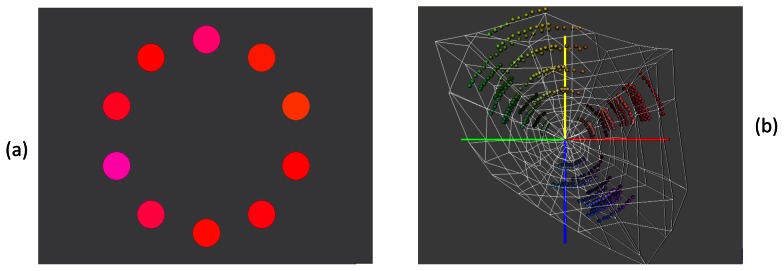
The modified hue selection task. (a) 2-deg colour patches (test stimuli) are presented at an eccentricity of 4 degrees. To obtain unique red (or green) the observer is asked to click on that patch that contains neither yellow nor blue. Similarly, to obtain unique yellow (or blue), an annulus consisting of yellowish (or bluish) patches is displayed and the observer is asked to click on that patch that is neither red nor green. In a previous experiment, we confirmed that the hue settings are not affected by the arrangement of the hue patches; randomised and ordered hue patches yield the same mean results. (b) The chromatic properties of the test stimuli are shown in a 3D solid in CIELUV space. The outermost white line indicates the gamut of our monitor. On a particular trial, hues of similar saturation and brightness were displayed in an annulus (see a). E.g. To obtain unique red, reddish hues lying on an arc in the CIELUV space, are displayed and the observer is asked to select that reddish hue that is neither yellow nor blue.

To obtain settings of the unique hues we used a modified hue selection task [6]. Patches of slightly different hues were arranged along an annulus at constant eccentricity (Figure 1a) and the task of the observer was to select a patch that contains neither yellow nor blue (to obtain unique red and green). Unique yellow (blue) was obtained by asking observers to select a patch that contains neither red nor green. Each patch had a diameter of 2° of visual angle and was presented at an eccentricity of 4°. Each unique hue was determined at nine combinations of different saturation and brightness levels (ranging from 8.5 cd/m^2^ to 60 cd/m^2^). The exact luminance values varied slightly between the different hues so as to maximise the available monitor gamut (Figure 1b). Each of these nine settings was repeated three times. In total, 360 test colours (4 unique hues×9 combinations of different saturation-brightness levels×10 colour patches per test) were displayed. On a particular trial, the patches were of the same saturation and brightness, and only differed in hue (for an example, see Figure 1a), in order to facilitate the task for the observer.

To obtain achromatic settings (neutral grey), the annulus consisted of greyish patches of a higher luminance (60 cd/m^2^) than the background and the observer was asked to select that patch that contained neither red nor green and, at the same, contained neither yellow nor blue. To obtain the achromatic settings, each trial consisted of two stages; first the greyish patches in the annulus contained small random modulations (around a theoretical achromatic point set to D65) along a reddish-greenish axis and the observer had to select that patch that contained neither red nor green. In the 2^nd^ stage, the red-green coordinates were set to the selection obtain in the 1^st^ stage and random modulations were along a yellow-bluish axis were added. In the 2^nd^ stage the observer selected that greyish patch that appeared neither yellow nor blue. Each trial was repeated three times.

Experiments for the three different viewing conditions were conducted on separate occasions. On each occasion the observer sat in the experimental booth for at least 10 minutes prior to the start of the experiment to ensure complete adaptation to the ambient illumination. Under the dark viewing conditions observers also performed the tri-vector Cambridge Colour Test [19]. Normal range was defined as thresholds lower than 100×10^−4^ *u*'*v*' units for the protan and deutan lines, and lower than 150×10^−4^ *u*'*v*' units for the tritan line. Observers with thresholds beyond these limits received a small fee and were excluded from further experiments. All data reported in the results section are therefore from a colour-normal sample.

### Chromatic specifications

To calculate the cone absorptions in the long-, medium-, and short-wavelength-sensitive cones, we used the 2-deg cone fundamentals derived by Stockman and Sharpe [20]. The cone fundamentals were then scaled in terms of luminance units using the new V*(λ) (available at www.cvrl.org) such that luminance equals the sum of the L and M cone absorptions (Figure S1b). Then the appropriate scaling factor (k_m_ = 683) was applied to V*(λ) to obtain candela units [21]. The phosphor spectra were measured at peak output (Figure S1a), upsampled to 1 nm spacing and multiplied with the scaled cone fundamentals to obtain the transformation from linearized RGB space to LMS space. All calculation are performed in LMS cone space; to visualise the unique hue settings in a chromaticity diagram, we transform the LMS coordinates to the Boynton-McLeod (BML) chromaticity coordinates [2], [22]. In the BML space, the x-axis corresponds to the difference between the L and M cone signals, normalised to luminance; the y-axis shows the modulation of the S cones, again normalised to luminance (Figure S1d).

### Estimating the cone weightings

Colour-opponent mechanisms were first mentioned by Hering [23] who proposed that any hue can be described in terms of its redness or greenness and its yellowness or blueness. Red and green are opposite hues because they cannot–under normal viewing conditions without retinal stabilisation-be elicited simultaneously by a single colour stimulus; the same is true for blue and yellow. Hering therefore postulated the existence of two colour-opponent channels coding red-green and yellow-blue sensations. Quantitative estimates of these two colour-opponent channels were first obtained by Jameson and Hurvich [24]–[26] using a hue cancellation technique. For example, when a subject adjusts the red and green component of a yellowish stimulus such that it contains neither red nor green, then the red-green opponent channel (RG) is at equilibrium; consequently a red-green opponent mechanism produces a zero response for this yellowish stimulus (for details see [6]): 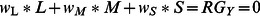
(1)


A yellowish stimulus with these differential L,M,S cone weights (w_L_,w_M_,w_S_) is void of any red and green, since it silences the putative red-green mechanism (i.e. RG_Y_ = 0); the stimulus is therefore referred to as ‘unique yellow’. Equation 1 defines a plane in three-dimensional LMS cone space. The vector (w_L_,w_M_,w_S_) is orthogonal to this plane and is called the normal vector for this plane; it characterises the chromatic properties of the red–green mechanism (RG_Y_). By definition, this mechanism is silenced by all yellowish colours on this plane and the plane is therefore the null plane for the RG_Y_ mechanism. We can derive an analogous equation for unique blue. A bluish stimulus that contains neither red nor green, produces a zero response in a red-green opponent channel: 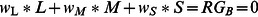
(2)


Unique red (Eq 3) and unique green (Eq. 4) are defined as colours that produce zero output in a yellow-blue opponent channel: 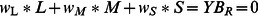
(3)

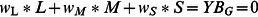
(4)


Altogether, we therefore estimate 12 coefficients, three for each unique hue, by a principal component analysis (PCA); the last eigenvector (explaining the least variance) is the normal vector. The length of the normal vector is normalised to unity (for details see [6]).

### The Lens model by Pokorny et al

**Figure 2:**
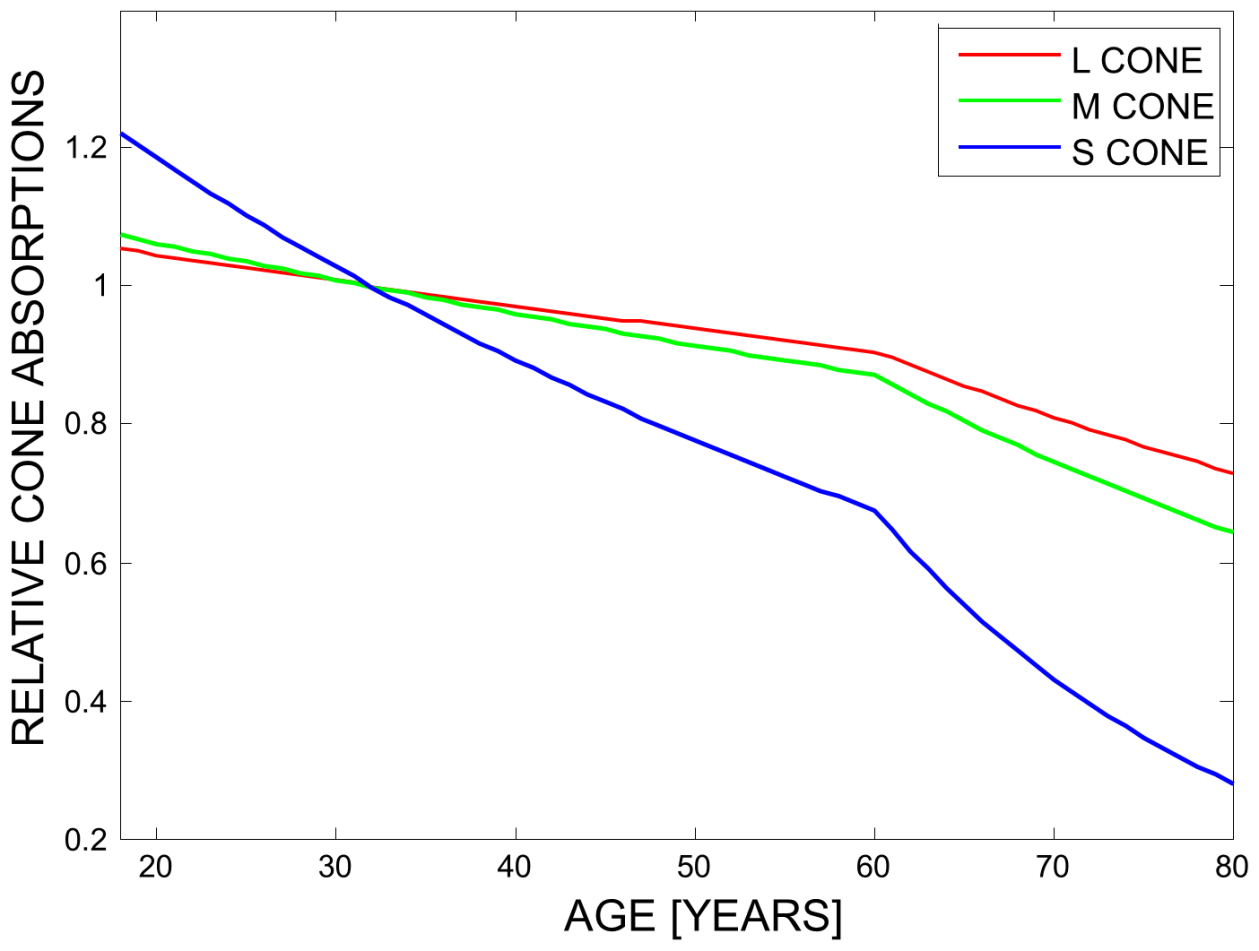
Lens transmission changes as a function of age. Relative L,M,S cone absorptions as a function of age were derived from the lens model by Pokorny et al [16]. To calculate the age-related relative loss in the three cone classes (L,M,S), the tabulated 2-deg cone fundamentals derived by Stockmann and Sharpe [20] were multiplied with the linear lens transmission (up to 650 nm), for each year of age. The resulting loss in the L,M,S cone signals a function of age is normalised to unity at 32 years of age.

We used the lens model by Pokorny et al [16] to predict the changes in lens transmission as a function of age. To calculate the age-related relative loss in the three cone classes (L,M,S), the tabulated 2-deg cone fundamentals derived by Stockman and Sharpe [20] were multiplied with the linear lens transmission (up to 650 nm), for each year of age (Figure S1c). The resulting loss in the L,M,S cone signals a function of age is normalised to unity at 32 years of age (Figure 2).

## Results

### 1. Effect of age on colour appearance

To evaluate whether hue perception changes with age and whether these changes are predicted by the age-related changes in the optical media, we proceed as follows.

(1) From the unique hue settings, we first estimate the cone weightings of the mechanisms that mediate the four unique hues (using Eq. 1–4; Experimental Procedures) and test whether these observed cone weightings change with age.

(2) We then predict the cone weightings using the lens model by Pokorny et al. [16]. By comparing the observed with the predicted cone weightings as a function of age, we can ascertain whether changes in hue perception are explained by the yellowing of the lens.

**Figure 3:**
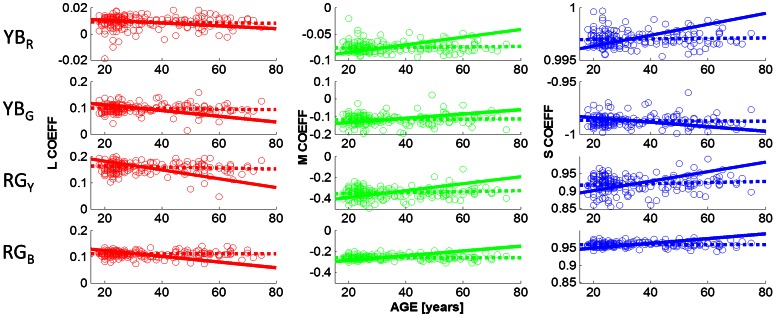
Cone weightings as a function of age. The cone weightings obtained under the dark viewing conditions are shown as a function of age. The L,M,S cone weightings (columns 1–3) of the four chromatic mechanisms (YB_R_, YB_G_, RG_Y_, RG_B_) that are silenced by unique red (row 1), green (row 2), yellow (row 3), and blue (row 4) are plotted as open symbols (see Eq 1–4 for details). Dashed lines (OBS) indicate the best fitting straight line to the data; solid lines (PRED) are the predictions based on the lens model (see Experimental Procedures). None of the observed slopes differs significantly from zero (p<0.05), indicating that the unique hue settings do not change with age. All slopes predicted from the lens model, however, differ from zero (p<0.001).

Figure 3 shows the observed cone weightings under the dark viewing conditions. The cone weightings (in columns: L,M,S) are plotted as a function of age, for the chromatic mechanisms associated with the four unique hues (Figure 3: in rows: YB_R_, YB_G_, RG_Y_, RG_B_). Observed cone weightings are shown by open circles; the best fitting regression lines for the observed cone weightings are indicated by dashed lines. For example, the first row shows the cone weightings of the chromatic mechanism that is silenced by unique red, denoted YB_R_ (cf Eq. 3); the leftmost graph shows the L cone weighting (w_L_) for this chromatic YB mechanism; the 2^nd^ column indicates the M cone weightings (w_M_) and the 3^rd^ column the S cone weightings (w_S_) of the mechanism that is silenced by unique red (YB_R_). Row 2 shows the cone weightings for the chromatic mechanism that is at equilibrium for unique green (YB_G_; cf. Eq 4); unique green is nulling a mechanism that takes the difference between the L and S cone signals, with very little M cone input. The mechanism that is silenced by unique red (YB_R_; cf Eq. 3) has a very different chromatic tuning; it has an L cone coefficient close to zero; indicating that this yellow-blue mechanism is parallel to the L cone plane and is primarily driven by the difference between the M cone and the S cone inputs. The observation that not a single linear mechanism is silenced by unique red and green confirms previous findings [6], [26].

Cone weightings for the mechanisms that are silenced by unique yellow and unique blue are shown in rows 3 and 4, respectively (cf. Eq. 1 and 2). As expected, the cone weightings for these two hues are very similar, consistent with the hypothesis that unique yellow and blue are generated by silencing a single, approximately linear mechanism [25], [26], that combines the difference between the L and M cone signals with an S cone input. Identical scales on the y-axes allow for a comparison of the spread of the cone weightings (cf row 3 and 4): for all three cone classes, the spread for unique blue is smaller than for unique yellow indicated that observers are able to make more precise settings for unique blue in comparison to unique yellow. Most importantly, cone weightings are invariant with age for all hues and all cone classes: the slope of the regression lines (dotted lines in Figure 3) do not differ significantly (p<0.05) from zero for any hue and for any cone class.

The solid lines in Figure 3 indicate the predicted cone weightings under the lens model (see Figure 2; Experimental Procedures). To arrive at these predictions, we adjusted the L,M,S cone co-ordinates of the hue settings to incorporate the age-dependent lens transmission. We then calculated the normal vectors for each hue (see Methods: Estimating the Cone Weightings) which reflects the chromatic properties of the mechanism that is being nulled by a particular hue. For example, for the chromatic mechanism that is silenced by unique red (denoted YB_R_ in Figure 3, row 1) the lens model predicts a greater S cone weighting with an increase in age, to stay at equilibrium. This is explained by the significant decrease in short-wavelength light passing through the lens when age increases (Figure 2, and Figure S1c). To compensate for the weaker S cone signal, this mechanism would need to increase the weighting of the S cone input. The discrepancy between the predicted and the observed regression lines reflect the amount of compensation of the hue mechanisms. All predicted regression lines differ significantly from zero, at p<0.001, whereas none of the observed slopes differs from zero. Slopes, coefficients of determination (R^2^), and the corresponding p-values are provided in Table 1 for all hues and all cone classes, for the observed cone weightings (OBS) and the predicted cone weightings (PRED). The first part of Table 1 describes the statistical results when stimuli were viewed under dark conditions.

**Table 1:** Dependency of cone weightings on Observer age.

			L COEFF			M COEFF			S COEFF		
ILLUM	HUE		SLOPE	R^2^	p	SLOPE	R^2^	p	SLOPE	R^2^	p
DARK	YB_R_	OBS	−1.57E-05	0.002	0.539	4.00E-05	0.003	0.426	2.73E-06	0.003	0.466
		PRED	−1.14E-04	0.118	0.000	7.17E-04	0.459	0.000	5.20E-05	0.501	0.000
	YB_G_	OBS	−8.34E-05	0.005	0.343	1.21E-04	0.003	0.435	−9.60E-06	0.001	0.726
		PRED	−1.06E-03	0.461	0.000	1.17E-03	0.256	0.000	−2.16E-04	0.267	0.000
	RG_Y_	OBS	−1.98E-04	0.012	0.138	4.17E-04	0.014	0.103	1.72E-04	0.011	0.152
		PRED	−1.67E-03	0.475	0.000	3.24E-03	0.481	0.000	1.36E-03	0.410	0.000
	RG_B_	OBS	−5.55E-07	0.000	0.993	3.40E-05	0.001	0.743	2.66E-06	0.000	0.938
		PRED	−1.09E-03	0.658	0.000	2.30E-03	0.728	0.000	6.76E-04	0.694	0.000
DAYLIGHT (D65)	YB_R_	OBS	−1.15E-05	0.002	0.564	2.84E-05	0.003	0.447	1.07E-07	0.000	0.973
		PRED	−1.10E-04	0.156	0.000	7.15E-04	0.573	0.000	5.18E-05	0.594	0.000
	**YB_G_**	OBS	−2.46E-04	0.031	0.016*	4.03E-04	0.026	0.028*	−6.25E-05	0.023	0.040*
		PRED	−1.16E-03	0.422	0.000	1.36E-03	0.238	0.000	−2.37E-04	0.261	0.000
	RG_Y_	OBS	−1.38E-04	0.004	0.379	2.99E-04	0.006	0.313	1.25E-04	0.003	0.430
		PRED	−1.65E-03	0.388	0.000	3.17E-03	0.398	0.000	1.37E-03	0.299	0.000
	RG_B_	OBS	4.76E-05	0.003	0.424	−5.32E-05	0.001	0.610	4.27E-06	0.000	0.895
		PRED	−1.05E-03	0.636	0.000	2.21E-03	0.710	0.000	6.47E-04	0.665	0.000
COOL WHITE FLOURSECENT (CWF)	YB_R_	OBS	−1.94E-05	0.004	0.367	2.78E-05	0.002	0.577	2.10E-06	0.002	0.541
		PRED	−1.17E-04	0.148	0.000	7.34E-04	0.540	0.000	5.30E-05	0.556	0.000
	YB_G_	OBS	−1.73E-04	0.018	0.069	2.52E-04	0.012	0.138	−3.24E-05	0.006	0.290
		PRED	−1.11E-03	0.448	0.000	1.25E-03	0.243	0.000	-2.32E-04	0.251	0.000
	RG_Y_	OBS	−1.42E-04	0.006	0.299	2.95E-04	0.007	0.251	1.06E-04	0.004	0.397
		PRED	−1.60E-03	0.443	0.000	3.10E-03	0.456	0.000	1.26E-03	0.377	0.000
	RG_B_	OBS	−4.54E-06	0.000	0.925	3.28E-05	0.001	0.699	5.64E-06	0.000	0.839
		PRED	−1.04E-03	0.700	0.000	2.20E-03	0.758	0.000	6.34E-04	0.729	0.000

Slopes, coefficients of determination (R^2^) and the p-values for observed and predicted cone weightings (L COEFF, M COEFF, S COEFF) for all hues and all ambient illumination conditions (DARK, DAYLIGHT, COOL WHITE FLOURESCENT). Under the null hypothesis all slopes are zero, indicating no age dependency. All predicted slopes differ from zero (p<0.001; rows labelled with PRED). Observed slopes (rows labelled with OBS) are significantly different from zero (p<0.05) only for the unique green settings under D65. For all other hues and ambient illumination settings we find no age-related changes in perceived hue.

**Figure 4:**
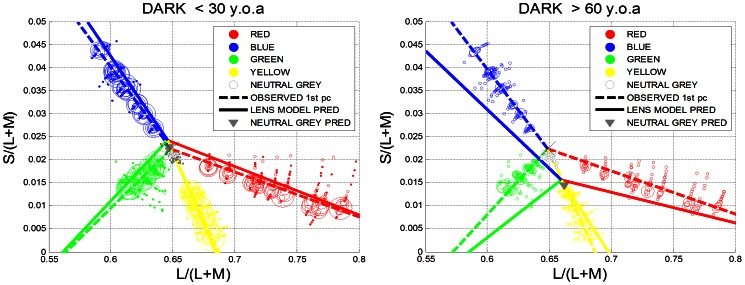
Comparison between the lower (<30 y.o.a.) and the upper age group (>60 y.o.a). (a) Observed unique hue settings for the dark viewing conditions are shown as circles in Boynton-MacLeod chromaticity diagram for the younger age group (<30 y.o.a). Symbol size is proportional to the number of observations per data point. Dotted lines indicate the observed unique hue lines (summarised by the 1^st^ principal component); the solid lines denote the predicted unique hue lines assuming the lens model by Pokorny et al.. The grey triangle is the average prediction for neutral grey settings, assuming the same lens model. For the younger age group, only very small hue shifts are expected assuming the lens model. All predictions are made with respect to a 32 old observer. (b). For the older age group (>60 y.o.a) large hue shifts are predicted under the lens model. The difference between the observed and predicted hue lines indicates the amount of compensation present in these hue judgements.

To visualise the amount of compensation, the observed and predicted unique hue settings are plotted in the Boynton-McLeod-Diagram for the lower (<30 y.o.a; Figure 4a) and the higher age group (>60 y.o.a; Figure 4b). The unique hues settings are summarised by the 1^st^ principal component: dashed lines indicate observed settings, and the solid lines are the predictions derived from the lens model. In the younger age group (Figure 4a) we expect little difference between the observed and the predicted hue settings since the optical density of the lens is not changing the transmission of short-wavelength light dramatically (Figure S1c).The older age group (>60 y.o.a.; Figure 4b), however, should experience significant hue shifts in the absence of compensatory mechanisms. E.g. blue hues should shift downwards in the BML diagram towards yellow-green hues; green is predicted to shift towards yellow. Achromatic settings (indicated by open grey symbols) are predicted to appear yellowish (indicated by a solid grey triangle in Figure 4b).

**Figure 5:**
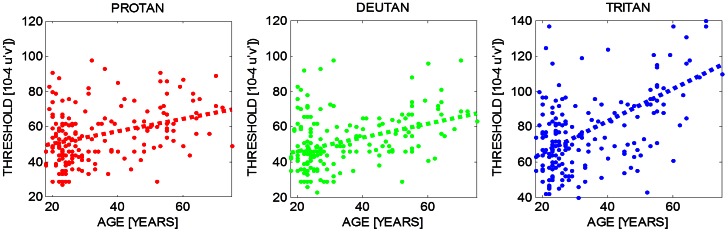
Chromatic sensitivity as a function of age. Thresholds along the protan (left most), deutan (middle) and tritan line (right most) is plotted as function of age for all 185 observers. A linear regression analysis revealed that the slope for all three sets of thresholds differs significantly from zero: Protan: slope = 0.004 R^2^ = 0.119 p<0.001; Deutan: slope = 0.003 R^2^ = 0.139 p<0.001; Tritan: slope = 0.254 R^2^ = 0.258 p<0.001.

While we find no evidence for age-related hue changes, the chromatic sensitivity, as assessed with the Cambridge Trivector Test [19] declines significantly (p<0.001) with age along the protan, the deutan, and the tritan line (Figure 5). The most robust age-related sensitivity changes are observed along the tritanopic confusion line (rightmost panel in Figure 5), as expected from the differential loss of short-wavelength light in the ageing lens (see also Figure S1c).

### 2. Effect of ambient illumination on unique hue settings for different age groups

**Figure 6:**
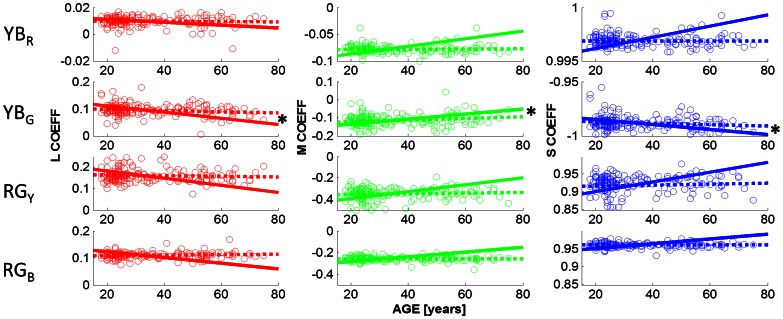
Cone weightings obtained under D65. Details are as in Figure 3. Under D65, the cone coefficients associated with unique green are age-dependent (p<0.05; for L,M,S cone coefficients). Observed slopes that differ significantly from zero are indicated with a star (*). All predicted slopes are different from zero (p<0.001). The direction of the observed age-related changes (dashed lines; row 2) are predicted by the lens model (solid lines), but not the magnitude (cf Table 1).

**Figure 7:**
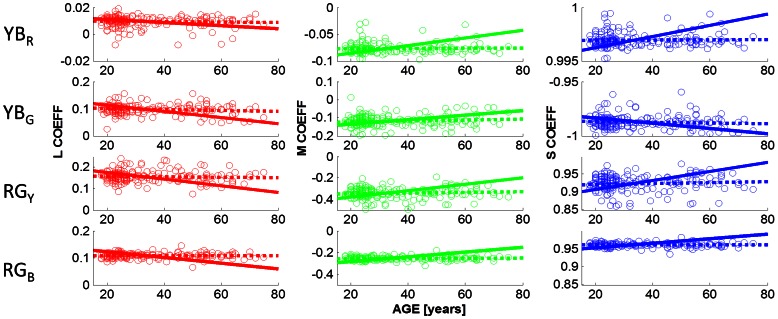
Cone weightings obtained under CWF. Details are as in Figure 1. As for the dark viewing condition, the observed coefficients do not depend on age (p>0.05), whereas the lens model predicts significant age-related changes (p<0.001).

Our second aim was to assess whether the adaptation mechanisms that underlie the hue constancy across age operate in a similar manner for different ambient viewing conditions. Figures 6 and 7 show the cone weightings obtained under ambient illumination condition close to D65 (daylight) and under cool white fluorescent lighting (CWF), respectively. In contrast to the dark viewing condition, green settings change slightly with age under adaptation to D65. Slopes, coefficients of determination and p-values are listed in Table 1 (Daylight (D65), row for YB_G_); observed slopes that differ significantly from zero are indicated with a star (*) in Table 1 and in Figure 6 (row 2: p<0.05 for L, M and S cone weightings). The direction of the age-related changes in the observed L,M,S coefficients (dashed lines) is predicted by the lens model (solid line) but not the magnitude. The lens model predicts greater changes in the coefficients than is observed in the data.

Under adaptation to CWF, the results mirror those for dark adaptation (Figure 7; Table 1, lower part): cone coefficients are independent of age (p>0.05 for all hue settings) whereas the lens model predicts highly significant age-related changes.

For all ambient illumination conditions the lens model predicts the direction of the observed changes but not the magnitude. Table 1 shows that the observed (OBS) and predicted slopes (PRED) as a function of age; in all cases the sign of the slope is the same; but only for the green settings under adaptation to daylight do these age-related hue changes reach statistical significance.

## Discussion

Our main finding is that colour appearance mechanisms are to large extent unaffected by the known age-related changes in the optical media (yellowing of the lens) whereas the ability to discriminate between small colour differences is compromised with an increase in age.

Werner and colleagues [14] suggested that the approximate hue constancy across the life span could be explained by a concurrent parallel decline in cone signals. A mechanism that takes the difference between the L and M cone signals, for example, will not be greatly affected by a decline in both the L and M cone outputs. We tested this hypothesis directly by calculating the cone weightings for each of the four unique hue mechanisms (Eq. 1–4) and then used a lens model to predict how perceived hues should change given the changes in the lens transmission for different wavelengths. The predictions for the hue changes expected under the lens model (Figure 2) are shown by solid lines in Figures 3, 6, and 7 (cf. Table 1 for statistical regression results). While the directions of the predictions are consistent with the data, e.g. for unique green (row 2 in figures 3, 6 and 7, the lens model predicts a decrease in the L and the S cone weightings and an increase in the M cone weightings, the magnitude of the observed hue changes is significantly smaller than the observed age-related cone weightings. We therefore conclude, in line with Webster et al. [27], that hue constancy across the life span is not a simple consequence of the differential cone combinations of the higher-order chromatic mechanisms

The second possibility is that the human visual system can adjust the cone weightings of the chromatic mechanisms over the lifespan and thereby compensate for a decline in peripheral cone signals. Our data are consistent with this hypothesis. The decline in sensitivity along the protan, deutan and tritan line we documented in the same set of observers (Figure 5) suggests that these compensatory mechanisms are likely to arise at a late, probably cortical site and that these higher-order appearance mechanisms operate in parallel to the discrimination mechanisms revealed in the chromatic sensitivity task. The decline in chromatic discrimination sensitivity is consistent with the deutan and tritan decline reported for the 50+age groups [28]–[30]

This dissociation between discrimination and appearance mechanisms is supported by Neitz and colleagues [31], [32] who showed that shifts in unique yellow induced by long-term changes in the chromatic environment are not due to receptoral or sub-cortical changes, but must be of cortical origin, probably after chromatic information from both eyes has been integrated. The question remains how these higher order colour mechanisms receive feedback on the strength of their cone inputs. Neitz et al's unique yellow data are consistent with the hypothesis that the gains of the L and M cones are adjusted such that the red-green opponent mechanism is at equilibrium for the average daylight [33], but this recalibration is by no means complete [34]. Our results provide further evidence that the brain uses information about the statistical properties of our chromatic environment to adjust the weighting of the receptor signals to achieve hue constancy across the life span.

Our second aim was to assess whether there is a differential age-related effect of ambient illumination on hue constancy. Comparing the cone weightings under the dark viewing condition (Figure 3) with those under adaptation to D65 (Figure 6) and CWF (Figure 7) reveals that the mean unique hue settings across the entire sample are not affected by the illumination conditions (see also Figure S2and S3). A closer look at the age dependency of the observed cone weightings (Table 1) shows that there is a differential effect of adaptation on hue constancy: only under daylight (D65) adaptation do the age-related changes in the green settings reach statistical significance (Table 1; Figure 6, row 2); what appears uniquely green for young observers appears more yellowish for older observers. Older observers require more S cone input to achieve unique green when the settings are obtained under simulated daylight, but still much less than predicted by the lens model. This is consistent with the idea that the yellow-blue mechanism (YB_G_) which is silenced by the unique green settings is most affected by the yellowing of the lens (Eq. 2); [35]). Similar conclusions were reached by Okajima & Takase [36] who assessed age-related colour changes using Munsell Colour chips instead of self-luminous lights.

If the visual system were able to fully compensate for the changes in the optical media, the observed cone weightings should not vary with age. We find that, under most of the tested conditions, this is the case; only under adaptation to daylight (D65), green hues change slightly with age.

In summary, we find evidence for compensatory mechanisms operating on higher-order colour functions and thereby ensuring that hue remains approximately constant despite the known age-related changes in the lens. The concurrent age-related decline in the chromatic discrimination sensitivity suggests that the neural site of these compensatory mechanisms is probably cortical; the underlying mechanism is still poorly understood, but is consistent with the idea that it is based on invariant sources in our visual environment.

## Supporting Information

Figure S1 **Spectra, cone fundamentals and lens model.** (a) The Spectral radiance distribution of the CRT phosphors b) The Stockman-Sharpe cone fundamentals used to obtain the lens predictions (c) Lens transmission as a function of wavelength, based on the Pokorny et al. lens model. Each line indicates the transmission of a particular age group, from 20 to 80 y.o.a. From top to bottom. (d). The Boynton-MacLeod Coordinates derived from the Stockmann-Sharpe cone fundamentals.
(TIF)

Figure S2 **Hue settings for the lower and the upper age group under D65.** Details as in Figure 4 (a) Observed unique hue settings for D65 are shown as circles in Boynton-MacLeod chromaticity diagram for the younger age group (<30 y.o.a). Symbol size is proportional to the number of observations per data point. Dotted lines indicate the observed unique hue lines (summarised by the 1st principal component); the solid lines denote the predicted unique hue lines assuming the lens model by Pokorny et al. (1987). The grey triangle is the average prediction for neutral grey settings, assuming the same lens model. For the younger age group, only very small hue shifts are expected assuming the lens model. All predictions are made with respect to a 32 old observer. (b). For the older age group (>60 y.o.a) large hue shifts are predicted under the lens model.
(TIF)

Figure S3 **Hue settings for the lower and the upper age group under CWF.** Details as in Figure 4. (a) Observed unique hue settings under CWF are shown as circles in Boynton-MacLeod chromaticity diagram for the younger age group (<30 y.o.a). Symbol size is proportional to the number of observations per data point. Dotted lines indicate the observed unique hue lines (summarised by the 1st principal component); the solid lines denote the predicted unique hue lines assuming the lens model by Pokorny et al. (1987). The grey triangle is the average prediction for neutral grey settings, assuming the same lens model. For the younger age group, only very small hue shifts are expected assuming the lens model. All predictions are made with respect to a 32-year old observer. (b). For the older age group (>60 y.o.a) large hue shifts are predicted under the lens model.
(TIF)

## References

[1] Helmholtz H (2000) Treatise on Physiological Optics (1896): Thoemmes.

[2] Derrington AM, Krauskopf J, Lennie P (1984) Chromatic mechanisms in lateral geniculate nucleus of macaque. Journal of Physiology 357: 241–265. 6512691 10.1113/jphysiol.1984.sp015499 PMC1193257

[3] Gegenfurtner KR, Kiper DC, Levitt JB (1997) Functional properties of neurons in Macaque area V3. Journal of Neurophysiology 77: 1906–1923. 9114244 10.1152/jn.1997.77.4.1906

[4] Barbur JL, Harlow AJ, Plant GT (1994) Insights Into the Different Exploits of Color in the Visual-Cortex. Proceedings of the Royal Society of London Series B-Biological Sciences 258: 327–334. 10.1098/rspb.1994.0181 7886066

[5] Hurlbert A (2003) Colour vision: primary visual cortex shows its influence. Current Biology 13: 270. 10.1016/s0960-9822(03)00198-2 12676104

[6] Wuerger SM, Atkinson P, Cropper SJ (2005) The cone inputs to the unique-hue mechanisms. Vision Research 45: 3210–3223. 16087209 10.1016/j.visres.2005.06.016

[7] Valberg A (1971) A method for the precise determination of achromatic colours including white. Vision Research 11: 157–160. 5551494 10.1016/0042-6989(71)90231-8

[8] DeValois RL, Devalois KK, Switkes E, Mahon L (1997) Hue scaling of isoluminant and cone-specific lights. Vision Research 37: 885–897. 9156186 10.1016/s0042-6989(96)00234-9

[9] Webster MA, Miyahara E, Malkoc G, Raker VE (2000) Variations in normal color vision. I. Cone-opponent axes. Journal of the Optical Society of America A 17: 1535–1544. 10.1364/josaa.17.001535 10975363

[10] Webster MA, Miyahara E, Malkoc G, Raker VE (2000) Variations in normal color vision. II. Unique hues. Journal of the Optical Society of America A 17: 1545–1555. 10.1364/josaa.17.001545 10975364

[11] Webster MA, Webster SM, Bharadwaj S, Verma R, Jaikumar J, et al (2002) Variations in normal color vision. III. Unique hues in Indian and United States observers. Journal of the Optical Society of America a-Optics Image Science and Vision 19: 1951–1962. 10.1364/josaa.19.001951 12365615

[12] Mollon JD (2009) A neural basis for unique hues? 19: R441–R442. 10.1016/j.cub.2009.05.008 19515347

[13] Stoughton CM, Conway BR (2008) Neural basis for unique hues. Current Biology 18: R698–R699. 18727902 10.1016/j.cub.2008.06.018

[14] Werner JS (1996) Visual problems of the retina during ageing: Compensation mechanisms and colour constancy across the life span. Progress in Retinal and Eye Research 15: 621–645.

[15] Schefrin BE, Werner JS (1990) Loci of spectral unique hues throughout the life span. Journal of the Optical Society of America A 7: 305–311. 10.1364/josaa.7.000305 2299452

[16] Pokorny J, Smith VC, Lutze M (1987) Aging of the human lens. Applied Optics 26: 1437–1440. 20454339 10.1364/AO.26.001437

[17] Werner A, Bayer A, Schwarz G, Zrenner E, Paulus W (2010) Effects of ageing on postreceptoral short-wavelength gain control: transient tritanopia increases with age. Vision Research 50: 1641–1648. 20457174 10.1016/j.visres.2010.05.004

[18] Peters A (2007) The Effects of Normal Aging on Nerve Fibers and Neuroglia in the Central Nervous System; Riddle D, editor: CRC Press. 21204349

[19] Regan BC, Reffin JP, Mollon JD (1994) Luminance noise and the rapid determination of discrimination ellipses in colour deficiency. Vision Research 34: 1279–1299. 8023437 10.1016/0042-6989(94)90203-8

[20] Stockman A, Sharpe LT (2000) Spectral sensitivities of the middle- and long-wavelength sensitive cones derived from measurements in observers of known genotype. Vision Research 40: 1711–1737. 10814758 10.1016/s0042-6989(00)00021-3

[21] Wyszecki G, Stiles WS (1982) Color Science: concepts and methods, quantitative data and formulae. New York: John Wiley & Sons.

[22] MacLeod DIA, Boynton RM (1979) Chromaticity diagram showing cone excitation by stimuli of equal luminance. Journal of the Optical Society of America 69: 1183–1186. 490231 10.1364/josa.69.001183

[23] Hering E (1964) Outlines of a theory of the light sense. Hurvich LM, translator. Cambridge, Massachusetts: Harvard University Press.

[24] Jameson D, Hurvich L (1955) Some quantitative aspects of an opponent-colors theory. I. Chromatic responses and spectral saturation. Journal of the Optical Society of America 45: 546–552.

[25] Larimer J, Krantz D, Cicerone C (1974) Oponenent-process additivity. I: Red/green equilibria. Vision Research 14: 1127–1140. 4428619 10.1016/0042-6989(74)90209-0

[26] Larimer J, Krantz D, Cicerone C (1975) Oponenent-process additivity. II: Yellow/blue equilibria and nonlinear models. Vision Research 15: 723–731. 1138490 10.1016/0042-6989(75)90291-6

[27] Webster MA, Halen K, Meyers AJ, Winkler P, Werner JS (2010) Colour appearance and compensation in the near periphery. Proceedings of the Royal Society B: Biological Sciences 277: 1817–1825. 20147325 10.1098/rspb.2009.1832 PMC2871866

[28] Paramei GV (2012) Color discrimination across four life decades assessed by the Cambridge Colour Test. J Opt Soc Am A 29: A290–A297. 10.1364/JOSAA.29.00A290 22330391

[29] Mateus C, Lemos R, Silva MF, Reis A, Fonseca P, et al (2013) Aging of Low and High Level Vision: From Chromatic and Achromatic Contrast Sensitivity to Local and 3D Object Motion Perception. PloS one 8: e55348. 23383163 10.1371/journal.pone.0055348 PMC3561289

[30] Knoblauch K, Saunders F, Kusuda M, Hynes R, Podgor M, et al (1987) Age and illuminance effects in the Farnsworth-Munsell 100-hue test. Appl Opt 26: 1441–1448. 20454340 10.1364/AO.26.001441

[31] Delahunt PB, Webster MA, Ma L, Werner JS (2004) Long-term renormalization of chromatic mechanisms following cataract surgery. Visual Neuroscience 21: 301–307. 15518204 10.1017/S0952523804213025 PMC2633455

[32] Neitz J, Carroll J, Yamauchi Y, Neitz M, Williams DR (2002) Color perception is mediated by a plastic neural mechanism that is adjustable in adults. Neuron 35: 783–792. 12194876 10.1016/s0896-6273(02)00818-8

[33] Mollon JD (1982) Color Vision. Annu Rev Psych 33: 41–85. 10.1146/annurev.ps.33.020182.000353 6977310

[34] Bompas A, Powell G, Sumner P (2013) Systematic biases in adult color perception persist despite lifelong information sufficient to calibrate them. Journal of Vision 13. 10.1167/13.1.19 23325346

[35] Schefrin BE, Werner JS (1990) Loci of Spectral Unique Hues Throughout the Life-Span. Journal of the Optical Society of America A-Optics Image Science and Vision 7: 305–311. 10.1364/josaa.7.000305 2299452

[36] Okajima K, Takase M (2001) Computerized simulation and chromatic adaptation experiments based on a model of aged human lens. Optical review 8: 64–70.

