# Supplementary figures and images for: Colour Constancy Across the Life Span: Evidence for Compensatory Mechanisms
Source: PLoS One. 2013 May 8;8(5):e63921. doi: 10.1371/journal.pone.0063921 (PMC3648508; doi:10.1371/journal.pone.0063921)

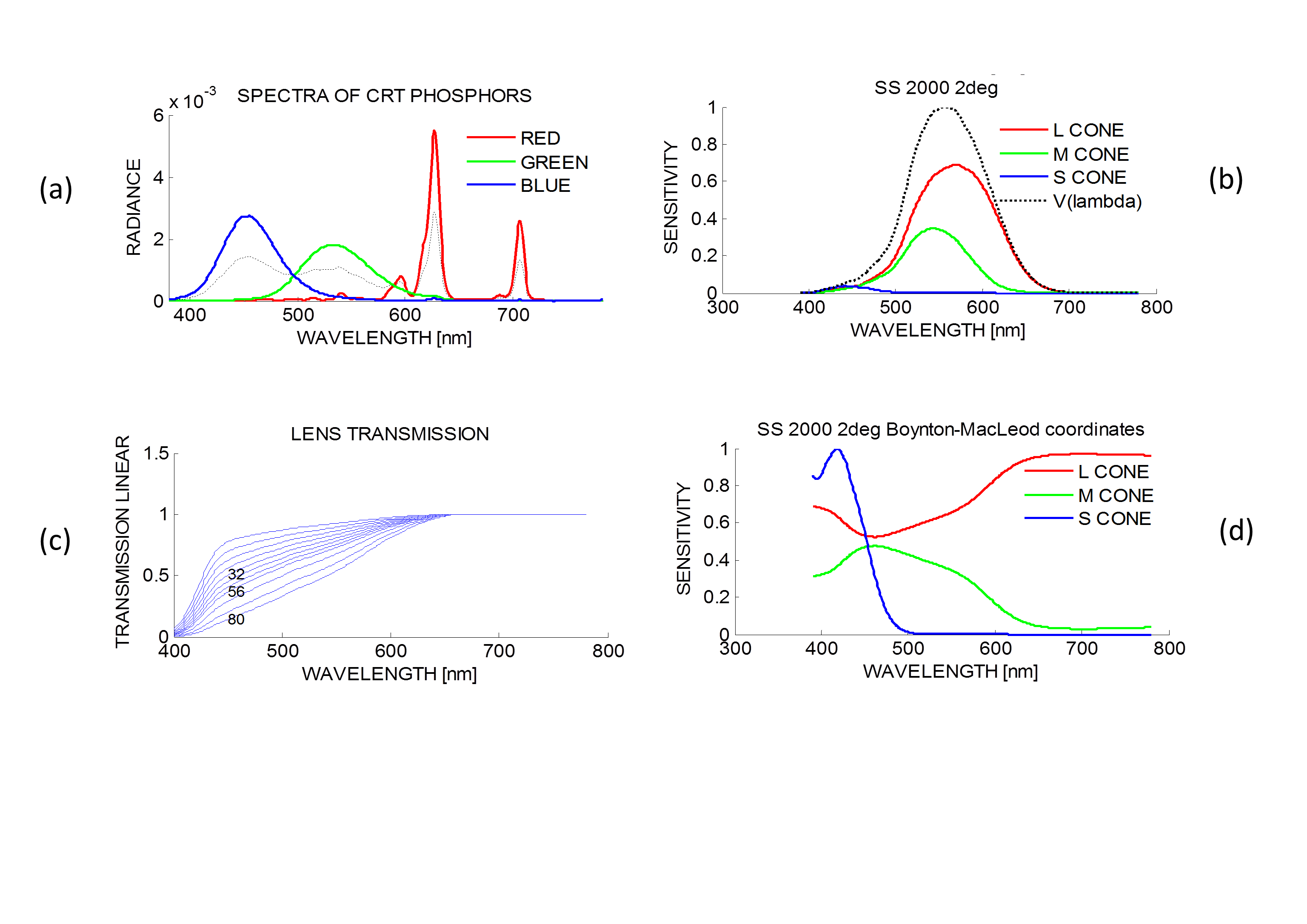

Supplement: Figure S1 — Spectra, cone fundamentals and lens model. (a) The Spectral radiance distribution of the CRT phosphors b) The Stockman-Sharpe cone fundamentals used to obtain the lens predictions (c) Lens transmission as a function of wavelength, based on the Pokorny et al. lens model. Each line indicates the transmission of a particular age group, from 20 to 80 y.o.a. From top to bottom. (d). The Boynton-MacLeod Coordinates derived from the Stockmann-Sharpe cone fundamentals. (TIF) [file pone.0063921.s001.tif]

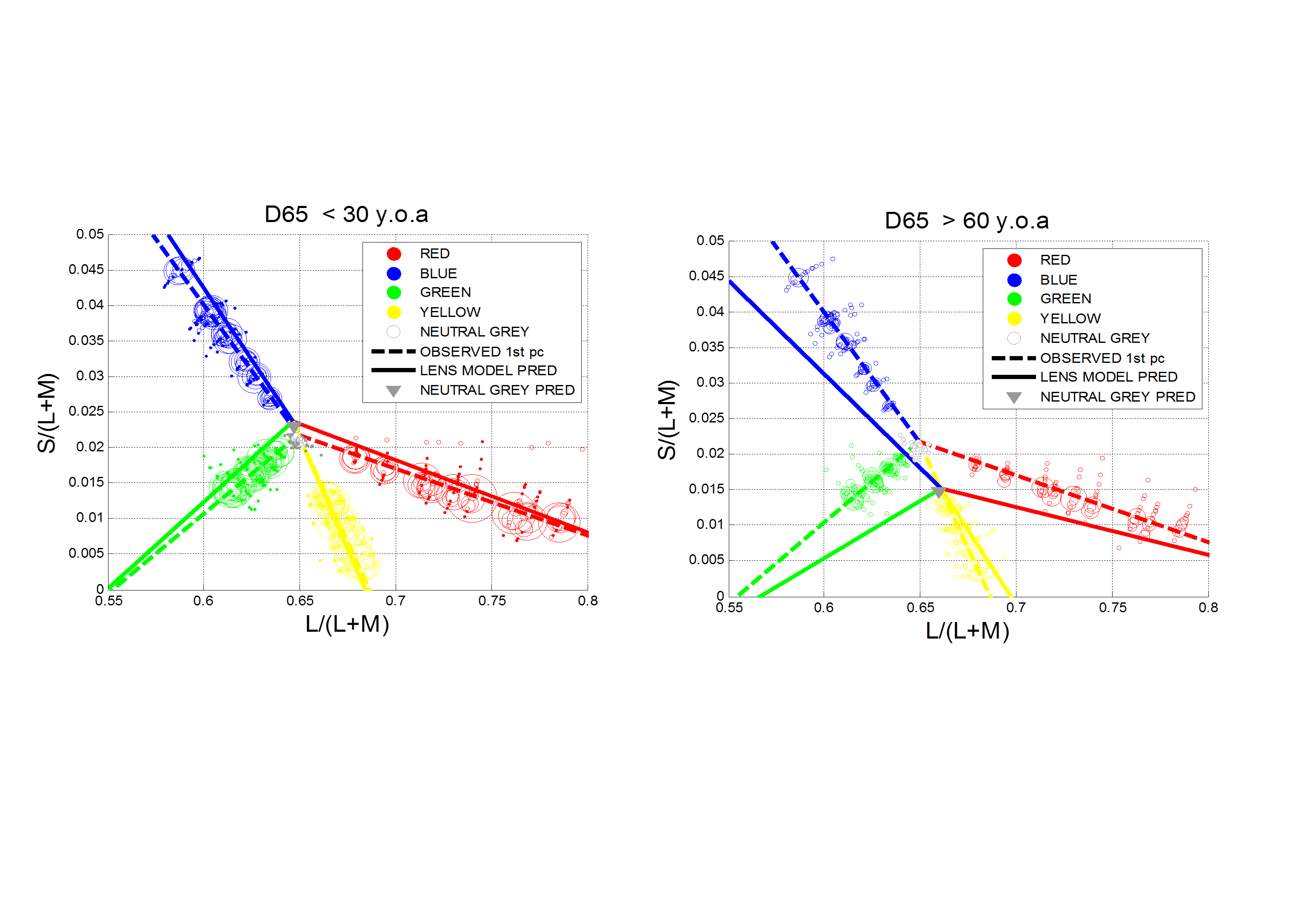

Supplement: Figure S2 — Hue settings for the lower and the upper age group under D65. Details as in Figure 4 (a) Observed unique hue settings for D65 are shown as circles in Boynton-MacLeod chromaticity diagram for the younger age group (<30 y.o.a). Symbol size is proportional to the number of observations per data point. Dotted lines indicate the observed unique hue lines (summarised by the 1st principal component); the solid lines denote the predicted unique hue lines assuming the lens model by Pokorny et al. (1987). The grey triangle is the average prediction for neutral grey settings, assuming the same lens model. For the younger age group, only very small hue shifts are expected assuming the lens model. All predictions are made with respect to a 32 old observer. (b). For the older age group (>60 y.o.a) large hue shifts are predicted under the lens model. (TIF) [file pone.0063921.s002.tif]

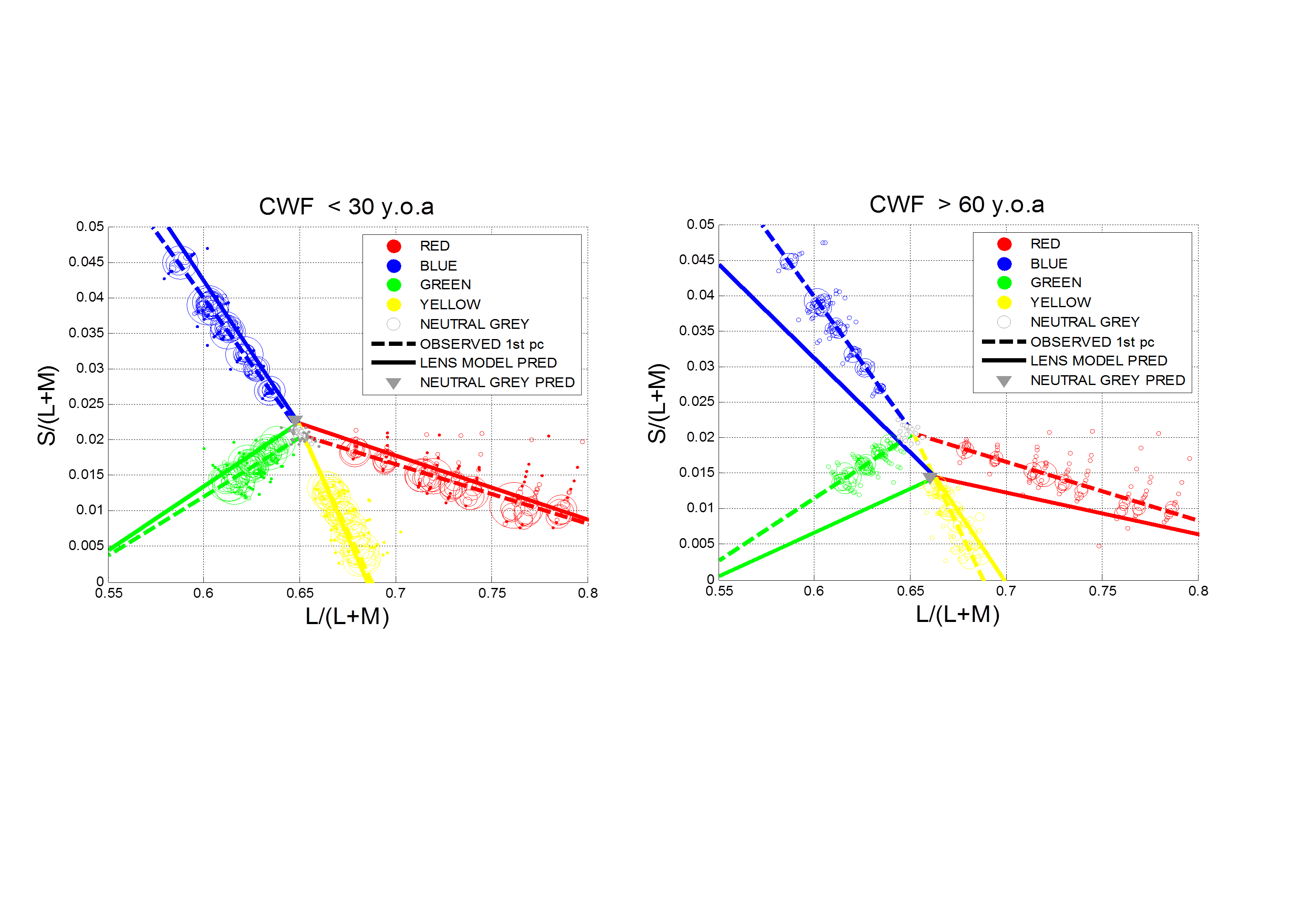

Supplement: Figure S3 — Hue settings for the lower and the upper age group under CWF. Details as in Figure 4. (a) Observed unique hue settings under CWF are shown as circles in Boynton-MacLeod chromaticity diagram for the younger age group (<30 y.o.a). Symbol size is proportional to the number of observations per data point. Dotted lines indicate the observed unique hue lines (summarised by the 1st principal component); the solid lines denote the predicted unique hue lines assuming the lens model by Pokorny et al. (1987). The grey triangle is the average prediction for neutral grey settings, assuming the same lens model. For the younger age group, only very small hue shifts are expected assuming the lens model. All predictions are made with respect to a 32-year old observer. (b). For the older age group (>60 y.o.a) large hue shifts are predicted under the lens model. (TIF) [file pone.0063921.s003.tif]
